# Both the C-Terminal Polylysine Region and the Farnesylation of K-RasB Are Important for Its Specific Interaction with Calmodulin

**DOI:** 10.1371/journal.pone.0021929

**Published:** 2011-07-05

**Authors:** Ling-Jia Wu, Li-Rong Xu, Jun-Ming Liao, Jie Chen, Yi Liang

**Affiliations:** State Key Laboratory of Virology, College of Life Sciences, Wuhan University, Wuhan, Hubei, China; University of Oulu, Germany

## Abstract

**Background:**

Ras protein, as one of intracellular signal switches, plays various roles in several cell activities such as differentiation and proliferation. There is considerable evidence showing that calmodulin (CaM) binds to K-RasB and dissociates K-RasB from membrane and that the inactivation of CaM is able to induce K-RasB activation. However, the mechanism for the interaction of CaM with K-RasB is not well understood.

**Methodology/Principal Findings:**

Here, by applying fluorescence spectroscopy and isothermal titration calorimetry, we have obtained thermodynamic parameters for the interaction between these two proteins and identified the important elements of K-RasB for its interaction with Ca^2+^/CaM. One K-RasB molecule interacts with one CaM molecule in a GTP dependent manner with moderate, micromolar affinity at physiological pH and physiologic ionic strength. Mutation in the polybasic domain of K-Ras decreases the binding affinity. By using a chimera in which the C-terminal polylysine region of K-RasB has been replaced with that of H-Ras and *vice versa*, we find that at physiological pH, H-Ras-(KKKKKK) and Ca^2+^/CaM formed a 1∶1 complex with an equilibrium association constant around 10^5^ M^−1^, whereas no binding reaction of K-RasB-(DESGPC) with Ca^2+^/CaM is detected. Furthermore, the interaction of K-RasB with Ca^2+^/CaM is found to be enhanced by the farnesylation of K-RasB.

**Conclusions/Significance:**

We demonstrate that the polylysine region of K-RasB not only contributes importantly to the interaction of K-RasB with Ca^2+^/CaM, but also defines its isoform specific interaction with Ca^2+^/CaM. The farnesylation of K-RasB is also important for its specific interaction with Ca^2+^/CaM. Information obtained here can enhance our understanding of how CaM interacts with K-RasB in physiological environments.

## Introduction

In mammals, the three classical *ras* genes encode four highly homologous proteins, H-Ras, N-Ras, and two isoforms K-RasA and K-RasB, and *ras* genes with point mutations are found in approximately 30% of human tumors [1], [2]. Ras proteins (21 kDa) are small GTPases, which cycle between inactive GDP-bound and active GTP-bound conformations at the plasma membrane, by interaction with a variety of guanine nucleotide exchange factors and GTPase activating proteins in response to stimulation. Following activation, Ras proteins bind and activate a plethora of downstream effector proteins and by this means control many cellular functions, including proliferation and differentiation [3], [4].

The Ca^2+^-binding protein calmodulin (CaM) is found in many eukaryotic cells. It is a small, heat- and acid-stable protein, whose amino acid sequence has been conserved almost perfectly throughout evolution. This protein mediates the control of a large number of enzymes by Ca^2+^. The control process occurs in two stages: binding of Ca^2+^ to CaM, accompanied by conformational changes, followed by the interaction of CaM with a variety of proteins [5]. Some Ras-related small G proteins such as Rin are found bound to the Ca^2+^/CaM complex [6], and CaM may regulate Rin activation in the Rin-mediated signaling pathway [7]. The Ras-related small G proteins Kir/Gem/Rad have also been found to bind to CaM [8], [9], and the binding of CaM to Kir and Gem significantly inhibits the binding of GTP to Kir/Gem [9]. Beguin *et al.* have addressed the mechanism of Kir/Gem regulation by CaM, and have indicated that the binding of Ca^2+^/CaM to Kir/Gem is required for inhibitory effect by promoting the cytoplasmic localization of Kir/Gem [10]. Furthermore, similar to Kir/Gem, CaM binding also regulates the subcellular distribution of Rad and Rem, both of which inhibit Ca^2+^ channel activity by preventing its expression on the cell surface [11], [12]. Wang and co-workers have shown that Ral-A is a CaM-binding protein, and Ca^2+^/CaM enhances the binding between GTP and Ral-A [13], [14]. Park *et al.* have reported that Ca^2+^/CaM can dissociate RalA from synaptic membranes [15]. It has been found that Ras-related small G protein Rab3A dissociates from synaptic membranes by forming a 1∶1 complex with Ca^2+^/CaM. CaM also plays a role in stimulating GTP binding to Rab3A that is complexed with GDP dissociation inhibitor, leading to the formation of an active GTP-bound form of the Rab3A/Ca^2+^/CaM complex [16], [17].

Agell's group has first reported the interaction of K-RasB and CaM [18], and has indicated that the phosphorylation of K-RasB by protein kinase C is inhibited by CaM [19]. CaM can cause dissociation of K-RasB from membranes [20], and the polybasic farnesyl domain of K-RasB can act as a target for Ca^2+^/CaM [21], [22]. Three different regions in K-RasB are important for the interaction: the hypervariable region (HVR), the α-helix between amino acids 151 and 166, and the switch II [23]. A growing body of literature has demonstrated that the HVR of K-RasB is responsible for its specific interactions with CaM [22]–[24], although interaction between K-RasB and CaM is not required for Golgi K-RasB translocation induced by Ca^2+^ influx in striatal neurons [23].

In this study, we employed several biophysical approaches, such as fluorescence spectroscopy and isothermal titration calorimetry (ITC), to characterize the interaction between K-RasB and Ca^2+^/CaM. We demonstrated that K-RasB interacted with Ca^2+^/CaM in a GTP dependent manner. Furthermore, our data show that both the C-terminal polylysine region of K-RasB and the farnesylation of K-RasB are important for its specific interaction with Ca^2+^/CaM.

## Materials and Methods

### Materials

GDP, GppNHp, farnesylpyrophosphate, and Ru(bpy)_3_Cl_2_ were purchased from Sigma (Sigma-Aldrich Co, St. Louis, MO). Anti-CaM monoclonal antibody was obtained from Boster (Wuhan, China). Ni nitrilotriacetic acid agarose was obtained from Qiagen GmbH (Hilden, Germany), and SP Sepharose Fast Flow, Q Sepharose Fast Flow, and Phenyl Sepharose 6 Fast Flow were Amersham Biosciences products (Uppsala, Sweden). All other chemicals used were made in China and were of analytical grade.

### Plasmids and proteins

The coding sequence of full-length K-RasB was amplified from pEGFP-C3-K-RasB, a kind gift from Prof. Yoav Henis (Department of Neurobiochemistry, Tel Aviv University). The amplified fragment was digested with Nde1/Xho1 and inserted into pET28a vector to generate pET28a-K-RasB. K-RasB mutations were obtained from pET28a-K-RasB by single PCR but with reverse and forward oligonucleotides carrying the appropriate mutations. We exchanged the C-terminal polylysine region of K-RasB-(175–180), KKKKKK, with the corresponding sequence of H-Ras-(175–180), DESGPC, and *vice versa*, to create two chimeric Ras proteins, K-RasB-(DESGPC) and H-Ras-(KKKKKK). K-RasB-(DESGPC) and H-Ras-(KKKKKK) were obtained from pET28a-K-RasB and pET28a-H-Ras by three rounds of PCR reaction but with reverse and forward oligonucleotides carrying the appropriate mutations. All resultant constructs were confirmed by sequencing.

Plasmids containing target sequences were transformed into *Escherichia coli* BL21 (DE3) strain. The expression of recombinant Ras proteins was induced by adding isopropyl-β-D-thiogalactopyranoside in the culture medium to a final concentration of 0.1 mM. The induction was permitted for 10 h at 25°C and cells were harvested by centrifugation at 8000 *g* for 10 min at 4°C. H-Ras was expressed and purified as described previously [25].

For K-RasB and its mutants, the harvested bacterial pellets from 2 liters of Luria Broth medium was resuspended and lysed in 100 ml of binding buffer (50 mM sodium phosphate buffer containing 500 mM NaCl, 1 mM MgCl_2_, 1 mM DTT, and 5 mM GDP, pH 7.8). The clarified supernatants were collected and applied to a Ni-nitrilotriacetic acid-agarose column equilibrated in the binding buffer. The proteins were eluted using elution buffer (50 mM sodium phosphate buffer containing 500 mM NaCl, 1 mM MgCl_2_, 1 mM DTT, and 5 mM GDP, pH 7.8) with a gradient of increasing imidazole concentration from 10 to 100 mM. Fractions containing K-RasB, as detected by SDS-PAGE and by UV absorbance, were pooled and diluted 10 times by 20 mM HEPES buffer containing 100 mM NaCl, 1 mM MgCl_2_, 1 mM dithiothreitol (DTT), and 5 mM GDP (pH 7.0), and applied to a SP-Sepharose column for K-RasB and its mutants. Proteins were eluted by 150–800 mM linear NaCl gradient. His tags were removed by thrombin. K-RasB was loaded with GTP analogue GppNHp by incubation with a 5-fold molar excess of GppNHp prepared as described [26] in the presence of 200 mM ammonium sulfate and 5 units of alkaline phosphatase per mg of protein at 30°C for 2 h. The concentrations of proteins were determined by the calculated extinction coefficient of 20 962 M^−1^ cm^−1^ for K-RasB and K-RasB mutants, and 22 452 M^−1^ cm^−1^ for H-Ras and H-Ras mutants, concerning the amino acid composition of each protein and the binding nucleotide.

CaM was purified from bovine brain (Hongxing, Wuhan, China) according to Gopalakrishna and Anderson [27], using Phenyl Sepharose affinity chromatography as the main purification step. The primary structure of bovine brain CaM is the same as that of human brain CaM. The concentration of CaM was determined by the extinction coefficient of 3006 M^−1^ cm^−1^ in its Ca^2+^ saturation state.

### *In vitro* farnesylation of Ras proteins

*Escherichia coli* DH5α strain containing yeast farnesyl transferase expressing plasmid PGP114-2/1/2 (a kind gift from Prof. Poulter, The University of Utah) was incubated in Super Broth (SB) medium to express farnesyl transferase [28]. The harvested bacterial pellets from 0.5 liter of SB medium was resuspended, lysed in 50 ml of lysis buffer (50 mM HEPES buffer containing 5 mM MgCl_2_, 40 mM NaCl, 10 µM ZnSO_4_, 1 mM phenylmethanesulfonyl fluoride, and 1 mM DTT, pH 7.5), and clarified by centrifuge. Purified *Escherichia coli* expressed Ras protein (5 mg), GDP (0.25 mg) and farnesylpyrophosphate (0.25 mg) were added to the clarified lysate and incubated overnight at room temperature. Ras proteins were re-purified from the lysate by the N-terminal hexahistidine tags and were separated from unprocessed Ras proteins by extraction with Triton X-114 [29], followed by the removal of the detergent with Q Sepharose column. About 2 mg of farnesylated Ras proteins were recovered from the original 5 mg of unprocessed starting material.

### Cross-linking reactions and Western blot analysis

Cross-linking reactions were carried out as described [30]. Briefly, 50 µM CaM with or without 50 µM K-Ras in a total volume of 18 µl in 20 mM HEPES buffer (pH 7.4) containing 150 mM NaCl, 1 mM CaCl_2_, 1 mM MgCl_2_, 2.5 mM ammonium persulfate, and 0.125 mM Ru(bpy)_3_Cl_2_ was placed in a 1.5-ml Eppendorf tube positioned for 1 min parallel to the beam of light from a 150-W common flashlight as the light source. Immediately after irradiation, samples were quenched with 6 µl of 4× loading buffer (250 mM Tris-HCl, pH 6.8, 8% SDS, 10 mM EDTA, 2.88 M β-mercaptoethanol, 40% glycerol, 2% Coomassie brilliant blue G 250, and 2% Phenol red), heated to 95°C for 5 min, and then applied to 13.5% SDS polyacrylamide gel. CaM was detected by using anti-CaM monoclonal antibody on Western blot.

### Isothermal titration calorimetry

ITC experiments on the interactions of CaM with K-RasB and its mutants in 20 mM HEPES buffer (pH 7.4) containing 150 mM NaCl, 1 mM CaCl_2_ and 1 mM MgCl_2_ were carried out at 25.0°C using an iTC_200_ titration calorimetry (MicroCal, Northampton, MA). A solution of 20–40 µM K-RasB protein was loaded into the sample cell (200 µl), and a solution of 0.3–0.8 mM CaM was placed in the injection syringe (40 µl). The first injection (1 µl) was followed by 18 injections of 2 µl. Dilution heats of CaM were measured by injecting CaM solution into buffer alone and were subtracted from the experimental curves prior to data analysis. The stirring rate was 600 rpm. The resulting data were fitted to a single set of identical sites model using MicroCal ORIGIN software supplied with the instrument, and the standard molar enthalpy change for the binding, 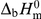, the dissociation constant, *K*_d_, and the binding stoichiometry, *n*, were thus obtained. The standard molar free energy change, 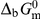, and the standard molar entropy change, 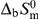, for the binding reaction were calculated by the fundamental equations of thermodynamics [31]:
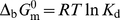
(1)

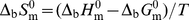
(2)


### Fluorescence measurements

Dansyl-CaM was prepared according to the method of Johnson *et al.* [32]. The bound dye concentration was determined by its absorbance at 335 nm using the molar extinction coefficient value of 3980 M^−1^ cm^−1^ [32]. Measurements of dansyl-CaM fluorescence were performed on an LS-55 luminescence spectrometer (PerkinElmer Life Sciences, Shelton, CT) at 25.0°C. A 0.5-ml solution containing 1 µM dansyl-CaM in 20 mM HEPES buffer (pH 7.4) containing 150 mM NaCl, 1 mM CaCl_2_ and 1 mM MgCl_2_ was titrated with 0.10–10 µl aliquots of K-RasB solution (500–100 µM) in the same buffer. Fluorescence spectra were recorded using an excitation wavelength of 335 nm and collecting the fluorescence emission between 420 and 560 nm. Slit widths were set at 5–10 nm.

The dissociation constant (*K*_d_) of a ligand to a protein can be calculated according to the following equation:
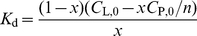
(3)where *C*_P,0_ is the total concentration of the protein (dansyl-CaM) being titrated, *C*_L,0_ is the total concentration of the ligand (K-RasB), and *x* represents the proportion of the protein bound by the ligand, which can be calculated according to the following equation:
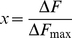
(4)where Δ*F* = *F*−*F*_0_, Δ*F*_max_ = *F*_max_−*F*_0_, and *F*_0_, *F* and *F*_max_ are the fluorescence intensities of dansylated CaM at 480 nm in the absence and in the presence of K-RasB and with saturating concentration of K-RasB.

Equations 3 and 4 are combined to deduce Equation 5:
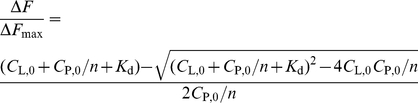
(5)


The dissociation constant, *K*_d_, and the binding stoichiometry, *n*, were determined by fitting Δ*F*/Δ*F*_max_ *versus C*_L,0_ to Equation 5. In this study, the total volume increase during titration was less than 5%, so the total concentration of dansyl-CaM was almost constant.

## Results

### K-RasB and Ca^2+^/CaM form a 1∶1 complex in a GTP dependent manner

**Figure 1:**
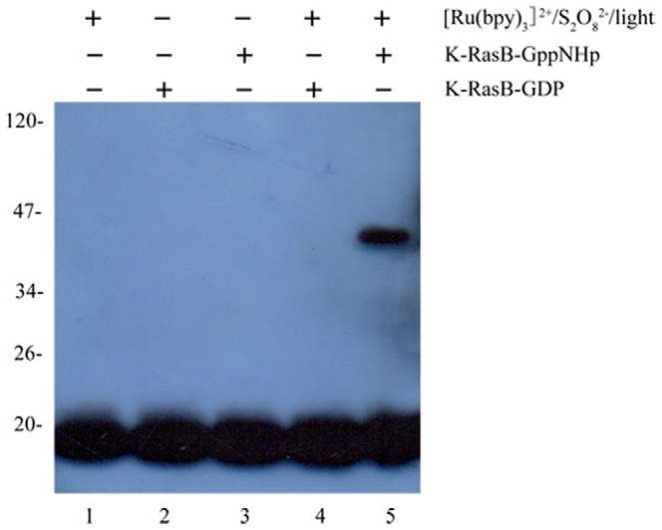
Photo-initiated cross-linking of the Ca^2+^/CaM with K-RasB-GDP and K-RasB-GppNHp. Samples containing Ca^2+^/CaM alone (lane 1), Ca^2+^/CaM and K-RasB-GDP (lane 4), or Ca^2+^/CaM and K-RasB-GppNHp (lane 5), were irradiated for 1 minute with a 150-W common flashlight as described in “Materials and Methods”. Samples containing Ca^2+^/CaM and K-RasB-GDP (lane 2), or Ca^2+^/CaM and K-RasB-GppNHp (lane 3) without Ru(bpy)_3_Cl_2_ and irradiation were also conducted as controls. Shown is a Western blot using antibody raised against CaM.

It has been reported that GTP-bound K-RasB interacts with CaM in a calcium dependent manner [18]. The photolysis of the ruthenium(II) tris-bipyridyl dication ([Ru(II)bpy_3_]^2+^) in the presence of ammonium persulfate was explored as a method to generate reactive intermediates that might bring about efficient cross-linking of associated proteins [30]. The bound of Ca^2+^/CaM to K-RasB was verified by chemical cross-linking using the photolysis of Ru(bpy)_3_Cl_2_. As shown in Fig. 1, photolysis resulted in the production of an extra band of about 45 kDa which was detected by anti-CaM antibody, corresponding to the covalently coupled 1∶1 complex of CaM with K-RasB-GppNHp (lane 5) whereas K-RasB-GDP not (lane 4).

**Figure 2:**
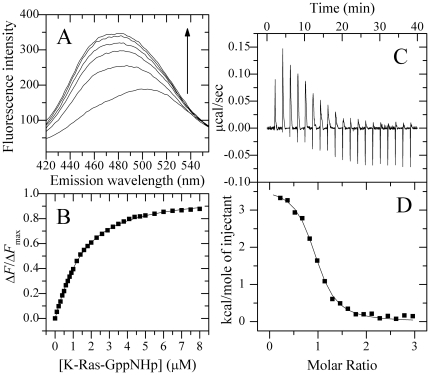
Interaction of K-RasB with Ca^2+^/CaM at 25.0°C. Fluorescence spectra (A) of 1.0 µM dansyl-CaM in the absence and in the presence of K-RasB-GppNHp at different concentrations. The arrow represents the concentration of K-RasB-GppNHp increases gradually from 0 (the bottom) to 8.0 µM (the top). Δ*F*/Δ*F*_max_ for the binding of K-RasB-GppNHp to Ca^2+^/CaM plotted as a function of the concentration of K-RasB-GppNHp (B). The solid squares were the experimental data and the solid line represented the best fit. The panel C represents typical calorimetric titration of K-RasB-GppNHp (20.0 µM) with CaM (300 µM) in the presence of 1 mM CaCl_2_. The panel D shows the plots of the heat evolved (kcal) per mole of CaM added, corrected for the heat of CaM, against the molar ratio of CaM to K-RasB. The data (solid squares) were fitted to a single set of identical sites model and the solid line represented the best fit. The corresponding parameters from B and D are summarized in Table 1.

The dansyl chomophore covalently bound to CaM is a sensitive probe for studying the interactions between CaM and its binding proteins [33]–[35]. To quantify the interaction between K-RasB and CaM, we carried out dansyl-CaM fluorescence titration experiments. As shown in Fig. 2A, the addition of K-RasB-GppNHp to Ca^2+^-saturated dansyl-CaM caused a significant increase in danysl fluorescence intensity and a pronounced blue shift of the fluorescence emission maximum from 504 to 479 nm, indicating that the dansyl group was located in a more hydrophobic environment in the complex than that in CaM alone. However no fluorescence change of dansyl-CaM was observed upon the addition of K-RasB-GppNHp in the absence of Ca^2+^, supporting the conclusion that the interaction between K-RasB and CaM was Ca^2+^ dependent (data not shown). As shown in Fig. 2B, about 90% of the saturation was reached when 8 µM K-RasB-GppNHp was added into Ca^2+^/CaM solution. The fluorescence titration data of Ca^2+^/CaM with K-RasB-GppNHp resulted in dissociation constants of 0.90±0.02 µM at physiological pH and physiologic ionic strength, as well as a 1∶1 CaM/K-RasB binding mode (Fig. 2B and Table 1).

**Table 1:** Thermodynamic parameters for the binding of Ras proteins to Ca^2+^/CaM as determined by ITC (*A*) and dansyl-CaM fluorescence titration (*B*) at 25.0°C.

Ras Proteins	*K*_d_ by *A* (µM)	*n*	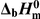 (kcal mol^−1^)	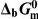 (kcal mol^−1^)	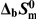 (cal mol^−1^ K^−1^)	*K*_d_ by *B* (µM)
K-RasB	1.10±0.12	0.924±0.013	3.63±0.72	−8.12±0.07	39.4±0.5	0.90±0.02
K-RasB-farn	0.39±0.16	1.13±0.01	3.33±0.11	−8.73±0.10	38.4±0.5	0.17±0.01
K-RasB-K175A	6.54±0.84	0.995±0.032	2.48±0.11	−7.07±0.08	32.0±0.7	4.26±0.07
K-RasB-K175A-farn	1.60±0.23	0.972±0.039	1.01±0.55	−7.90±0.09	29.9±2.0	1.02±0.03
H-Ras-(KKKKKK)	3.89±0.45	0.903±0.022	6.16±0.21	−7.38±0.07	45.4±1.0	2.53±0.18
H-Ras	NB	–	–	–	–	NB
K-RasB-(DESGPC)	NB	–	–	–	–	NB

The value of *n* was also determined as 1.0 for the binding of K-RasB to Ca^2+^/CaM by using Cross-linking.

NB, no binding observed in the present conditions.

Thermodynamic parameters, *K*_d_, 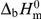, and *n*, were determined using a single set of identical sites model. The standard molar binding free energy (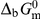) and the standard molar binding entropy (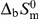) for the binding reaction were calculated using Equations 1 and 2 respectively. The buffer used was 20 mM HEPES buffer (pH 7.4) containing 150 mM NaCl, 1 mM CaCl_2_, and 1 mM MgCl_2_. Errors shown are standard errors of the mean.

ITC is thought to be one of the most reliable and high-precision methods to quantitate noncovalent, equilibrium protein interactions [36]–[38]. By using ITC, we obtained thermodynamic parameters for the interaction between Ca^2+^-saturated CaM and K-RasB proteins. Fig. 2C shows raw ITC curve resulting from the injections of Ca^2+^-saturated CaM into K-RasB-GppNHp solution. Fig. 2D shows the plots of the heat evolved per mole of CaM added, corrected for the heat of CaM dilution, against the molar ratio of CaM to K-RasB. The calorimetric data were best fit to a model assuming a single set of identical sites. The thermodynamic parameters for the binding of full-length K-RasB to Ca^2+^/CaM are summarized in Table 1. As shown in Table 1, one K-RasB molecule interacted with one CaM molecule in a GTP dependent manner with moderate, micromolar affinity at physiological pH and physiologic ionic strength. The binding reactions were driven entirely by large favorable increases in entropy. Our ITC and fluorescence data (Table 1) clearly indicated that at physiological pH, the GTP bound form of full-length K-RasB and Ca^2+^/CaM formed a 1∶1 complex with an equilibrium association constant around 10^6^ M^−1^.

### The polylysine region of K-RasB contributes importantly to the interaction of K-RasB with Ca^2+^/CaM

**Figure 3:**
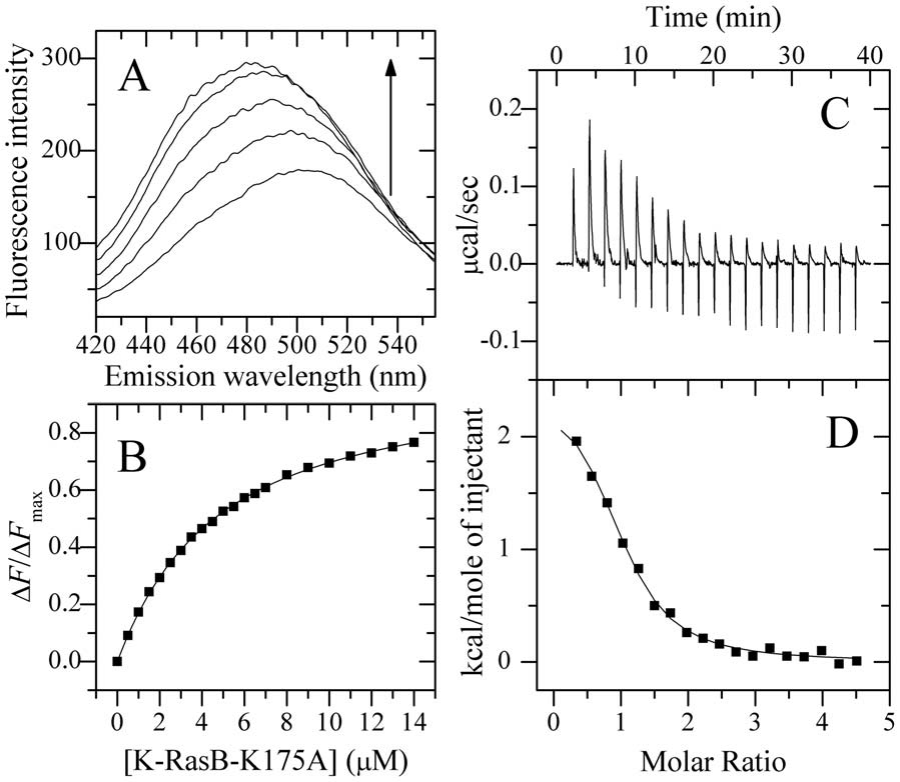
Interaction of K-RasB-K175A with Ca^2+^/CaM at 25.0°C. Fluorescence spectra (A) of 1.0 µM dansyl-CaM in the absence and in the presence of K-RasB-K175A-GppNHp at different concentrations. The arrow represents the concentration of K-RasB-K175A increases gradually from 0 (the bottom) to 14.0 µM (the top). Δ*F*/Δ*F*_max_ for the binding of K-RasB-K175A to Ca^2+^/CaM plotted as a function of the concentration of K-RasB-K175A (B). The solid squares were the experimental data and the solid line represented the best fit. The panel C represents typical calorimetric titration of K-RasB-K175A (35.0 µM) with CaM (800 µM) in the presence of 1 mM CaCl_2_. The panel D shows the plots of the heat evolved (kcal) per mole of CaM added, corrected for the heat of CaM, against the molar ratio of CaM to K-RasB-K175A. The data (solid squares) were fitted to a single set of identical sites model and the solid line represented the best fit. The corresponding parameters from B and D are summarized in Table 1.

As a general rule, CaM binding regions in proteins are characterized by the presence of several hydrophobic residues interspersed with a number of positively charged residues (lysine and arginine) [39]–[41]. To narrow down the key element of the interaction in the HVR of K-RasB, we evaluated the effects of lysine residues in the C-terminal polylysine region of K-RasB on the interaction between K-RasB and CaM by using dansyl-CaM fluorescence titration and ITC. As shown in Fig. 3, A and B, only 65% of the saturation was reached when 8 µM K-RasB-K175A-GppNHp was added into Ca^2+^-saturated dansyl-CaM solution, indicating that single mutation of a lysine residue, Lys-175, to alanine in C-terminal polylysine region of K-RasB resulted in a 6-fold decrease in the binding affinity. Fig. 3C shows raw ITC curves resulting from the injections of Ca^2+^-saturated CaM into K-RasB-K175A-GppNHp solution. Fig. 3D displays the plots of the heat evolved per mole of CaM added, corrected for the heat of CaM dilution, against the molar ratio of CaM to K-RasB-K175A. The calorimetric data were best fit to a single set of identical sites model. The thermodynamic parameters for the binding of K-RasB-K175A to Ca^2+^/CaM are summarized in Table 1. Clearly, at physiological pH, one K-RasB mutant molecule, interacted with one CaM molecule with markedly lower affinity than wild-type K-RasB (Table 1).

### The C-terminal polylysine region of K-RasB defines the specificity of the interaction of K-RasB with Ca^2+^/CaM

**Figure 4:**
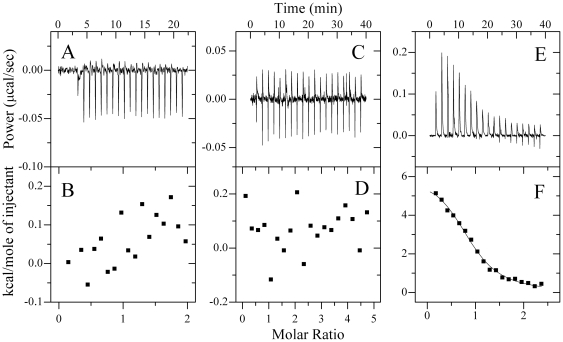
Interaction of H-Ras, K-RasB-(DESGPC), and H-Ras-(KKKKKK) with Ca^2+^/CaM at 25.0°C. The panels A, B, and C represent typical calorimetric titration of H-Ras-GppNHp (30.0 µM), K-RasB-(DESGPC)-GppNHp (25.0 µM) and H-Ras-(KKKKKK)-GppNHp (25.0 µM) with CaM (300 µM) respectively in the presence of 1 mM CaCl_2_. The panels D, E, and F show the plots of the heat evolved (kcal) per mole of CaM added, corrected for the heat of CaM, against the molar ratio of CaM to Ras proteins. The data (solid squares) were fitted to a single set of identical sites model and the solid line represented the best fit.

To better understand the role of the C-terminal polylysine region of K-RasB in the interaction between K-RasB and CaM, we exchanged the polylysine region of K-RasB-(175–180), KKKKKK, with the corresponding sequence of H-Ras-(175–180), DESGPC, and created two chimeric Ras proteins, K-RasB-(DESGPC) and H-Ras-(KKKKKK). The top panels in Fig. 4 representatively show raw ITC curves resulting from the injections of Ca^2+^-saturated CaM into GppNHp form of H-Ras (Panel A), K-RasB-(DESGPC) (Panel B) and H-Ras-(KKKKKK) (Panel C) solutions. The bottom panels in Fig. 4 show the plots of the heat evolved per mole of CaM added, corrected for the heat of CaM dilution, against the molar ratio of CaM to Ras proteins. The calorimetric data were best fit to a single set of identical sites model. The thermodynamic parameters for the binding of these two chimeric Ras proteins to Ca^2+^/CaM are summarized in Table 1. As shown in Table 1, at physiological pH, H-Ras-(KKKKKK) and Ca^2+^/CaM formed a 1∶1 complex with an equilibrium association constant around 10^5^ M^−1^. It has been reported that CaM binds to K-RasB, but not to H-Ras [18]. In the present study, we designed a K-RasB mutant unable to bind to CaM and thus defined the molecular determinants of CaM-K-RasB interaction. Similar to wild-type H-Ras, no binding reaction of K-RasB-(DESGPC) with Ca^2+^/CaM was detected by ITC and dansyl-CaM fluorescence titration (Table 1), demonstrating that the C-terminal polylysine region of K-RasB and the corresponding sequence of H-Ras, DESGPC, are the key sequences for CaM binding to K-RasB but not to H-Ras. The C-terminal polylysine region of K-RasB defines the specificity of the interaction of K-RasB with Ca^2+^/CaM.

### The farnesylation of K-RasB is important for its specific interaction with Ca^2+^/CaM

**Figure 5:**
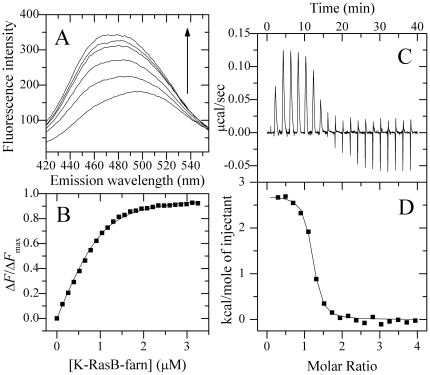
Interaction of farnesylated K-RasB with Ca^2+^/CaM at 25.0°C. Fluorescence spectra (A) of 1.0 µM dansyl-CaM in the absence and in the presence of GppNHp bound K-RasB-farn at different concentrations. The arrow represents the concentration of K-RasB-farn increases gradually from 0 (the bottom) to 3.3 µM (the top). Δ*F*/Δ*F*_max_ for the binding of K-RasB-farn to Ca^2+^/CaM plotted as a function of the concentration of K-RasB-farn (B). The solid squares were the experimental data and the solid line represented the best fit. The panel C represents typical calorimetric titration of K-RasB-farn (30.0 µM) with CaM (600 µM) in the presence of 1 mM CaCl_2_. The panel D shows the plots of the heat evolved (kcal) per mole of CaM added, corrected for the heat of CaM, against the molar ratio of CaM to K-RasB. The data (solid squares) were fitted to a single set of identical sites model and the solid line represented the best fit. The corresponding parameters from B and D are summarized in Table 1.

**Figure 6:**
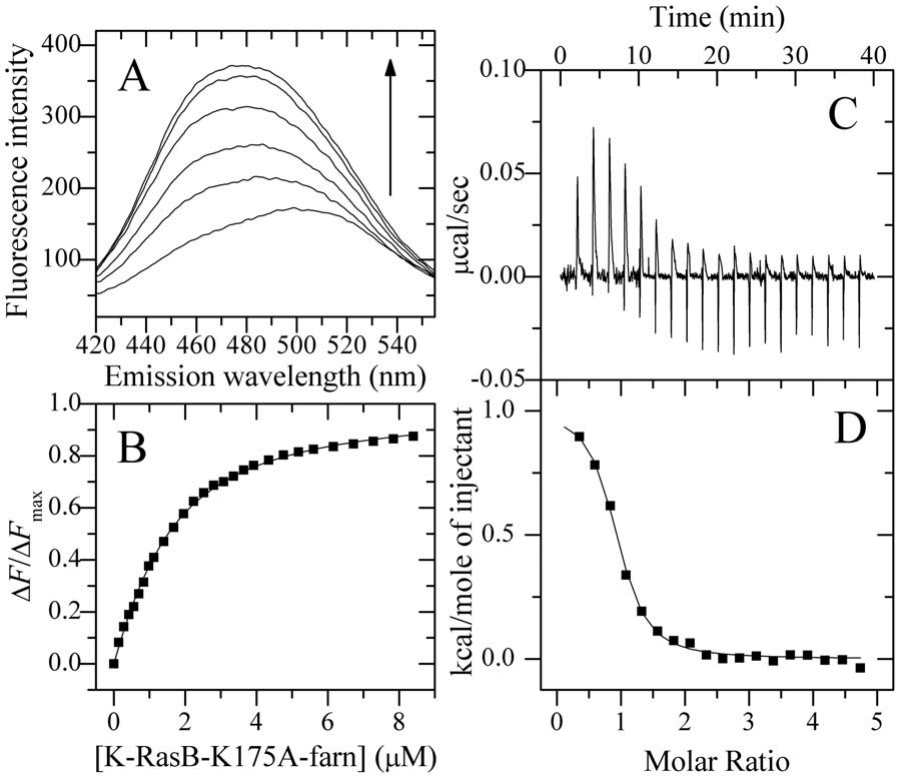
Interaction of farnesylated K-RasB-K175A with Ca^2+^/CaM at 25.0°C. Fluorescence spectra (A) of 1.0 µM dansyl-CaM in the absence and in the presence of GppNHp bound K-RasB-K175A-farn at different concentrations. The arrow represents the concentration of K-RasB-K175A-farn increases gradually from 0 (the bottom) to 8.4 µM (the top). Δ*F*/Δ*F*_max_ for the binding of K-RasB-K175A-farn to Ca^2+^/CaM plotted as a function of the concentration of K-RasB-K175A-farn (B). The solid squares were the experimental data and the solid line represented the best fit. The panel C represents typical calorimetric titration of K-RasB-K175A-farn (25.0 µM) with CaM (600 µM) in the presence of 1 mM CaCl_2_. The panel D shows the plots of the heat evolved (kcal) per mole of CaM added, corrected for the heat of CaM, against the molar ratio of CaM to K-RasB. The data (solid squares) were fitted to a single set of identical sites model and the solid line represented the best fit. The corresponding parameters from B and D are summarized in Table 1.

Plasma membrane association of Ras proteins requires posttranslational modification by C-terminal farnesylation. Here, we prepared fanesylated K-RasB *in vitro* and investigated the role of farnesylation of K-RasB in its interaction with Ca^2+^/CaM by fluorescence titration and ITC. As shown in Figs. 5A and 6A, titration of K-RasB-farn or K-RasB-K175A-farn into Ca^2+^-saturated dansyl-CaM caused a significant increase in danysl fluorescence intensity and a pronounced blue shift of the fluorescence emission maximum from 498 (or 499) to 471 (or 474) nm. As shown in Figs. 5B and 6B, the fluorescence titration data of Ca^2+^/CaM with K-RasB-farn and K-RasB-K175A-farn resulted in dissociation constants of 0.17±0.01 and 1.02±0.03 µM, respectively, 5- and 4-fold increases in the binding affinity compared with their unprocessed precursors (Figs. 5B and 6B and Table 1).

Figs. 5C and 6C respectively show raw ITC curves resulting from the injections of Ca^2+^-saturated CaM into K-RasB-farn and K-RasB-K175A-farn solutions. Figs. 5D and 6D show the plots of the heat evolved per mole of CaM added, corrected for the heat of CaM dilution, against the molar ratio of CaM to K-RasB. The calorimetric data were best fit to a model assuming a single set of identical sites. The thermodynamic parameters for the binding of K-RasB-farn and K-RasB-K175A-farn to Ca^2+^/CaM are also summarized in Table 1. According to the ITC data, K-RasB-farn and K-RasB-K175A-farn bound to Ca^2+^/CaM with 3- and 4-fold increases in the binding affinity compared with their unprocessed precursors, which is consistent with the fluorescence titration data. Clearly, the farnesylation of K-RasB is important for its specific interaction with Ca^2+^/CaM (Table 1).

## Discussion

It has been reported that the binding of K-RasB to Ca^2+^/CaM is specific and only K-RasB but not other isoforms of Ras proteins interacts with Ca^2+^/CaM [22]–[24]. Therefore, there may be sequences lying in HVR of H-Ras that inhibit the interaction with Ca^2+^/CaM, while the corresponding sequence of K-RasB not. In the present study, we demonstrated that the polylysine region of K-RasB not only contributed importantly to the interaction of K-RasB with Ca^2+^/CaM, but also defined its isoform specific interaction with Ca^2+^/CaM.

CaM is a ubiquitous protein that can regulate a number of different eukaryotic enzymes in a variety of cellular locations [42], [43]. It is a dumb bell shaped protein molecule with two EF-hand motifs connected by a small antiparallel β-sheet between the two loops. Calcium binding results in the exposure of two hydrophobic pockets for target protein binding, surrounded by negatively charged residues [44], [45]. Although the sequence identity among the CaM target sequences is low, all of the consensus peptides for such sequences posses common features, including their ability to form amphipathic α-helices containing several positively charged residues [43], [44]. In other words, CaM target sequences are characterized by the presence of several positively charged residues such as lysine. The present study demonstrated that the C-terminal polylysine region of K-RasB contributes importantly to the interaction of K-RasB with Ca^2+^/CaM and that H-Ras mutant containing such a polylysine region is able to bind to Ca^2+^/CaM. Therefore, this polylysine region could be a new CaM target sequence. Several studies have demonstrated that the positively charged residues in CaM binding proteins interact strongly with the negatively charged residues in CaM [40], [41]. Our work continues the story of the possible role of electrostatic interactions in forming protein-CaM complexes. We found a strong salt concentration dependence of the dissociation constant of K-RasB binding to CaM as determined by dansyl-CaM fluorescence titration at 25.0°C (Table 2). At physiologic ionic strength (150 mM), the equilibrium association constant was determined to be around 10^6^ M^−1^, and the data at 1.0 M NaCl afforded the nonionic contribution [46]. As shown in Table 2, the binding affinity of K-RasB for CaM was found to decrease significantly with increasing salt concentration, and the equilibrium association constant was smaller than 10^5^ M^−1^ as detected by dansyl-CaM fluorescence titration when NaCl concentration increased up to 1.0 M, suggesting that long-range electrostatic interactions between the polylysine region of K-RasB and the negatively charged residues in CaM are important but not essential for the interaction between K-RasB and CaM.

**Table 2:** Salt concentration dependence of the dissociation constants of K-Ras K-Ras-farn binding to CaM as determined by dansyl-CaM fluorescence titration at 25.0°C.

[NaCl] (M)	*K*_d_ (µM)	
	K-RasB	K-RasB-farn
0.10	0.52±0.02	0.15±0.01
0.15	0.90±0.02	0.17±0.01
0.20	1.11±0.10	0.25±0.03
0.30	1.80±0.22	0.36±0.06
0.50	5.68±0.54	0.78±0.08
0.70	21.4±2.2	1.47±0.16
1.0	49.5±3.0	2.05±0.19

The buffer used was 20 mM HEPES buffer (pH 7.4) containing 0.10–1.0 M NaCl, 1 mM CaCl_2_, and 1 mM MgCl_2_. Errors shown are standard errors of the mean.

Plasma membrane association of Ras proteins requires posttranslational modification such as geranylgeranylation [47] and C-terminal farnesylation. Here we demonstrated that the farnesylation of K-RasB is important for its specific interaction with Ca^2+^/CaM. The contribution of the farnesylation of K-RasB to the interaction became big in the presence of high concentrations of salt. As shown in Table S1, with increasing salt concentration, the binding affinity of unprocessed K-RasB for CaM decreased significantly but that of farnesylated K-RasB for CaM decreased less significantly, once again demonstrated that the farnesylation of K-RasB is important for its specific interaction with Ca^2+^/CaM.

The fate of K-RasB is different from that of the other isoforms such as H-Ras because of the unique presence of a polybasic, lysine-rich region in its HVR. Such a region is responsible for the stable association of K-RasB to the inner plasma membrane [48]. Potenza *et al.* [48] have indicated that the K-RasB-mediated signals required for the completion of embryonic development can also be transduced by H-Ras, whereas the physiology of the cardiovascular system seems to specifically require a K-RasB generated signal, highlighting a unique role for K-RasB in cardiovascular homeostasis. Because the first 85 amino acids of all of the Ras isoforms are identical and covers the site of interaction with all known Ras effectors [49], the unique role of K-RasB could due to its localization and activity regulation different from the other isoforms. It has been reported that CaM binds to K-RasB, but not to H-Ras, modulating its downstream signaling [18], causing dissociation of only K-RasB from membranes in a Ca^2+^-dependent manner that could result in its translocation to distinct regions of the cell and activation of diverse signaling pathways [20]. CaM inhibits K-RasB phosphorylation at Ser 181 and consequently modulates the functionality of both the wild type and the oncogenic form of K-RasB [50]. We demonstrated for the first time that the C-terminal polylysine region of K-RasB is the molecular determinant for CaM binding to K-RasB but not to H-Ras. Such a polylysine region could play an important role in the novel biological function of K-RasB to control cardiovascular homeostasis.

In conclusion we have shown that: (i) one K-RasB molecule interacts with one CaM molecule with moderate, micromolar affinity in physiological conditions, and the binding is GTP dependent; (ii) polylysine region of K-RasB contributes importantly to the interaction of K-RasB with Ca^2+^/CaM; (iii) the polylysine region of K-RasB defines the specificity of the interaction of K-RasB with Ca^2+^/CaM; (iv) the fanesylation of K-RasB increases the binding affinity of K-RasB for Ca^2+^/CaM. Information obtained here can enhance our understanding of how CaM interacts with K-RasB in physiological environments.

## Supporting Information

Table S1 **Salt concentration dependence of the dissociation constants of K-Ras K-Ras-farn binding to CaM as determined by dansyl-CaM fluorescence titration at 25.0°C.** The buffer used was 20 mM HEPES buffer (pH 7.4) containing 0.10–1.0 M NaCl, 1 mM CaCl_2_, and 1 mM MgCl_2_. Errors shown are standard errors of the mean.
(DOC)
