# Supplementary material for: Both the C-Terminal Polylysine Region and the Farnesylation of K-RasB Are Important for Its Specific Interaction with Calmodulin
Source: PLoS One. 2011 Jul 5;6(7):e21929. doi: 10.1371/journal.pone.0021929 (PMC3130059; doi:10.1371/journal.pone.0021929)
Supplement: Table S1 — Salt concentration dependence of the dissociation constants of K-Ras K-Ras-farn binding to CaM as determined by dansyl-CaM fluorescence titration at 25.0°C. The buffer used was 20 mM HEPES buffer (pH 7.4) containing 0.10–1.0 M NaCl, 1 mM CaCl2, and 1 mM MgCl2. Errors shown are standard errors of the mean. (DOC) [file pone.0021929.s001.doc]

| [NaCl] (M) | *K*_d_ (μM) | |
| --- | --- | --- |
|  | K-RasB | K-RasB-farn |
| 0.10 | 0.52 ± 0.02 | 0.15 ± 0.01 |
| 0.15 | 0.90 ± 0.02 | 0.17 ± 0.01 |
| 0.20 | 1.11 ± 0.10 | 0.25 ± 0.03 |
| 0.30 | 1.80 ± 0.22 | 0.36 ± 0.06 |
| 0.50 | 5.68 ± 0.54 | 0.78 ± 0.08 |
| 0.70 | 21.4 ± 2.2 | 1.47 ± 0.16 |
| 1.0 | 49.5 ± 3.0 | 2.05 ± 0.19 |
